# Slippery liquid infused porous surface (SLIPS) condensers for high efficiency air gap membrane distillation

**DOI:** 10.1038/s44172-025-00348-y

**Published:** 2025-03-15

**Authors:** Yashwant S. Yogi, Harsharaj B. Parmar, Hamid Fattahi Juybari, Sina Nejati, Akshay K. Rao, Rishav Roy, Mojtaba Zarei, Longnan Li, Soumyadip Sett, Abhimanyu Das, Nenad Miljkovic, Justin A. Weibel, David M. Warsinger

**Affiliations:** 1 School of Mechanical Engineering and Birck Nanotechnology Center, Purdue University, West Lafayette, IN 47907 USA; 2 Department of Mechanical Science and Engineering, University of Illinois, Urbana, IL 61801 USA; 3 Materials Research Laboratory, University of Illinois, Urbana, IL 61801 USA; 4 Department of Electrical and Computer Engineering, University of Illinois, Urbana, IL 61801 USA; 5 International Institute for Carbon Neutral Energy Research (WPI-I2CNER), Kyushu University, 744 Motooka, Nishi-ku, Fukuoka, 819-0395 Japan; 6 Air Conditioning and Refrigeration Center, University of Illinois at Urbana–Champaign, Urbana, IL 61801 USA; 7 Institute for Sustainability, Energy and Environment (iSEE), University of Illinois at Urbana-Champaign, Urbana, IL USA

**Keywords:** Energy modelling, Materials science, Mechanical engineering

## Abstract

To address growing water scarcity, we must improve the energy efficiency of thermal desalination technologies such as air gap membrane distillation. However, promising functional materials such as slippery liquid infused porous surfaces have not yet implemented for any desalination technology. Here, we fabricate and test slippery liquid infused porous surfaces (using Krytox 16,256 lubricant and CuO nanostructures) in an air gap membrane distillation apparatus. System-level transport models, validated by experimental data, establish a framework for improving performance through enhanced condensation surfaces. Results are obtained across a range of temperatures (50–80 °C), salinities (5–105 g/kg), and module lengths. We find that small air gap thickness and efficient droplet shedding significantly improves performance. The CuO Krytox process achieves these with a conductive-self-limiting coating, high nanostructure rugosity, strong covalent and metallic bonding, high hydrophobicity, minimal droplet pinning sites, and ultra-low contact angle hysteresis. The greatest efficiency enhancement from SLIPS is derived from the improved droplet shedding, which allows for reduced gap sizes without flooding, and is further augmented by the increased permeate flux.

## Abstract (web summary)

Thermal desalination processes such as air gap membrane distillation (AGMD) contribute to addressing water scarcity. Yashwant Yogi and colleagues test slippery liquid infused porous surfaces in the condensation plate of an AGMD apparatus. Models validated against experimental data suggest that AGMD energy efficiency could be boosted through the incorporation of such hydrophobic condensation surfaces, leading to improved droplet shedding.

## Introduction

Freshwater demand has grown rapidly because of the groundwater depletion, glacial melting, population increase, global desertification and climate change^1,2^. Desalination using thermal energy is a key solution for water scarcity, and has unique advantages for higher salinities and can be sustainable when using waste heat^3,4^.

**Fig. 1:**
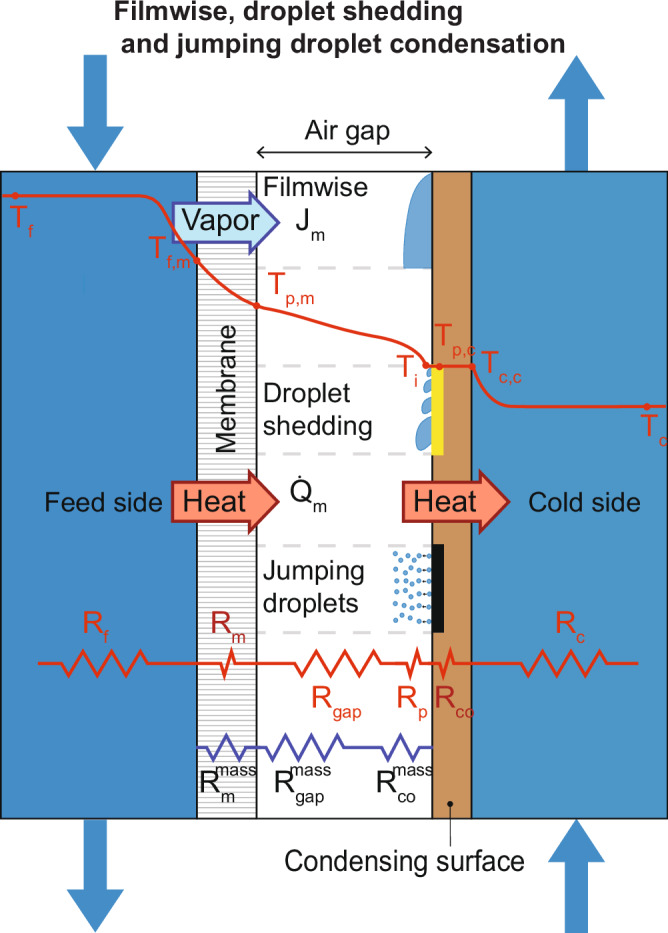
Schematic of air gap membrane distillation (AGMD). describing filmwise condensation on an untreated copper surface, droplet shedding on SLIPS and jumping droplet condensation on superhydrophobic surfaces. Thermal boundary layers, as well as heat and mass transfer resistances, are visualized across the membrane distillation (MD) module. ${{{\rm{J}}}}_{{{\rm{m}}}}$ is flux, ${{{\rm{T}}}}_{{{\rm{f}}}},{{{\rm{T}}}}_{{{\rm{f}}},{{\rm{m}}}},{{{\rm{T}}}}_{{{\rm{p}}},{{\rm{m}}}},{{{\rm{T}}}}_{{{\rm{i}}}},{{{\rm{T}}}}_{{{\rm{p}}},{{\rm{c}}}},{{{\rm{T}}}}_{{{\rm{c}}},{{\rm{c}}}},{{\rm{and}}}$ ${{{\rm{T}}}}_{{{\rm{c}}}}$ are temperatures of each part of the module, ${\dot{{{\rm{Q}}}}}_{{{\rm{m}}}}$ is heat transfer, ${{{\rm{R}}}}_{{{\rm{f}}}},{{{\rm{R}}}}_{{{\rm{m}}}},{{{\rm{R}}}}_{{{\rm{gap}}}},{{{\rm{R}}}}_{{{\rm{p}}}},{{{\rm{R}}}}_{{{\rm{co}}}},{{\rm{and}}}$ ${{{\rm{R}}}}_{{{\rm{c}}}}$ are heat transfer resistances, and ${{{\rm{R}}}}_{{{\rm{m}}}}^{{{\rm{mass}}}},{{{\rm{R}}}}_{{{\rm{gap}}}}^{{{\rm{mass}}}},{{\rm{and}}}$ ${{{\rm{R}}}}_{{{\rm{co}}}}^{{{\rm{mass}}}}$ are mass transfer resistances of different parts of the module.

Membrane distillation (MD) is an emerging thermal desalination technology with higher efficiencies than commercial methods like humidification dehumidification (HDH) and thermal vapor compression (TVC) desalination^5–11^. MD resembles a counter-flow heat exchanger with a hydrophobic, nanoporous membrane in between the hot and cold channel wall^5,12^. The temperature difference between the two sides of the membrane induces a vapor pressure difference, which causes water vapor to evaporate and condense on opposite sides of the liquid-blocking membrane (Fig. 1). MD can operate at low temperatures ( ~ 80 °C) and use waste heat from industries, power plants^13^, geothermal^14^ and solar^15,16^. MD can perform well across a diverse range of feedwater compositions^17^, membrane structures and materials^12^, cooling load^18^, and resist water with high fouling potential^19–22^.

MD design is heavily driven by tradeoffs between enhancing heat and mass transfer between the evaporation and condensing portions. Air gap membrane distillation (AGMD) the most common configuration, minimizes conductive heat losses with an air gap between the membrane and condensing plate^22–25^. Other configurations, like permeate gap membrane distillation (PGMD) or conductive gap membrane distillation (CGMD) use water flooded gaps to minimize the mass transfer resistance of water vapor (Fig. 1, blue resistors), but consequently have increased conductive heat losses (Fig. 1, red resistors) between the hot and cold sides^12,22^. Thus, these flooded gap configurations have lower thermal efficiencies, and tend to have lower overall efficiency, or gained output ratio (GOR), especially at high salinity ^26^. If the air gap in AGMD fully floods, then its performance resembles these configurations. To minimize flooding, air gaps larger than the capillary length of water ( > 2 mm) are usually used^10,27,28^, though optimization studies show that smaller gaps ( < 0.5 mm) are energy optimal^18^.

**Fig. 2:**
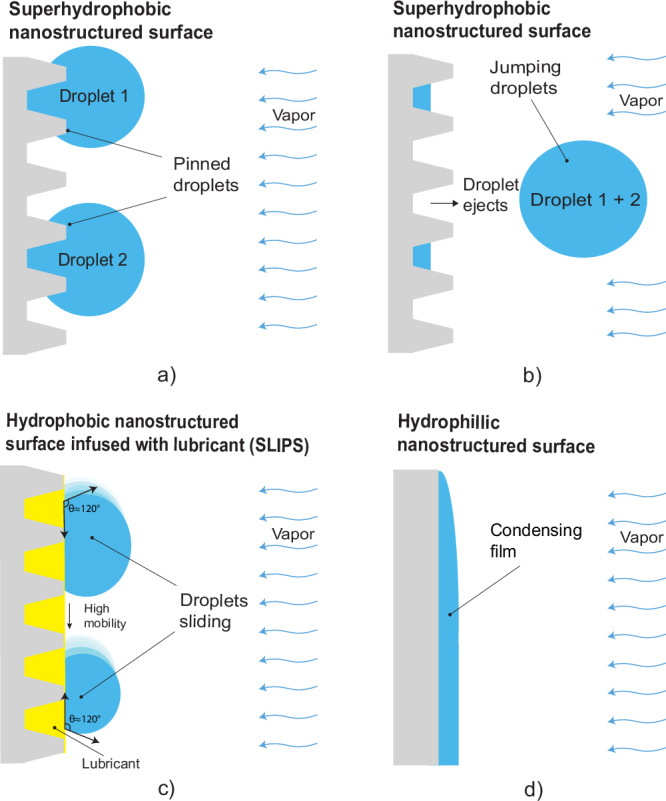
Comparison of condensation on different surfaces. **a** superhydrophobic surface prior- and **b** subsequent to the coalescence of droplets, **c** hydrophobic surface with lubricant infused into the porous structure (SLIPS), and **d** an untreated surface with filmwise condensation. The superhydrophobic surface exhibits non-wetting properties due to the higher contact angle, the SLIPS, characterized by a very low hysteresis contact angle (HCA < 2°), was found both slippery and non-wetting, while the untreated surface is wettable.

For many processes including MD, condensing on hydrophobic or superhydrophobic surfaces may induce dropwise condensation^29^. These enhanced regimes of condensation facilitate shedding of small droplets and thereby make more area available for condensation on the surface. Dropwise condensation has been shown to have five to seven times higher heat transfer coefficients than filmwise condensation^27,30–33^. In addition to regular dropwise condensation, jumping droplet condensation (Fig. 2b) on superhydrophobic surfaces has been shown to cause rapid droplet removal and enhance permeate flux up to 2 times in AGMD^28,34,35^. Enhanced mass transfer through superhydrophobic surfaces has also been well described through analytical models^29,36^, and can improve overall MD performance^22,37^. However, despite improved condensation rates, superhydrophobic surfaces promote continuous dropwise condensation only in optimal conditions. This regime breaks down with high vapor influx, often resulting in the coalescence of condensate droplets into a liquid film, which reduces heat transfer coefficients and overall MD performance^28,38^.

Other condensation enhancement methods that have shown mass transfer benefits in AGMD, include using wicking spacers^39^, thin air gaps, porous condenser surfaces^37^, and vertical module orientation^22^.

Lubricant-infused surfaces (LIS) and slippery lubricant-infused porous surfaces (SLIPS) are other promising enhanced condensation approaches that have not yet been implemented in thermal desalination. This class of surfaces uses liquid infused within porous structures (Fig. 2c) to cause both droplet shedding and self-cleaning^33,40^. SLIPS has been widely used for condensation due to its super slippery interface, both on flat^41^ and rough^42^ surfaces. There are two types of LIS surfaces: hydrophilic SLIPS or hydrophobic SLIPS. Hydrophilic SLIPS has shown a superior condensation performance than hydrophobic SLIPS in most cases^43^. Moreover, the stability of SLIPS strongly depends on the formation of wrapping layers surrounding the water droplets^44,45^. It is important to carefully assess whether to use hydrophilic or hydrophobic SLIPS for future studies, or to consider an alternative such as quasi-liquid surfaces^46^, which are non-LIS surfaces and tend to be more durable. We are leaning towards using hydrophobic SLIPS surfaces due to our previous research^47,48^. The low surface tension of the infused lubricant and the low surface energy of the porous or structured surface makes SLIPS surfaces hydrophobic, with the contact angle of water droplets around 120°. Moreover, the liquid-liquid interface (between the lubricant and water droplets) minimizes droplet pinning and increases droplet mobility, leading to ultra-low contact angle hysteresis ( < 2°) and faster droplet shedding respectively^44,49^. The ultra-low contact angle hysteresis on SLIPS results in much lower droplet sizes and droplet departure diameter, which indirectly supports higher nucleation density, improved droplet shedding and higher rates of re-nucleation when compared to most superhydrophobic surfaces^50–52^. Therefore, SLIPS have excellent potential for preventing flooding and increasing the efficiency of AGMD systems, compared to standard metallic surfaces for condensation.

In this study, we aim to improve the condensation heat transfer of AGMD by employing SLIPS. To understand and optimize SLIPS performance enhancement, we vary the air gap thicknesses, hot feed temperature, temperature difference, and feed salinities. To determine the optimal air gap thickness at different salinities, we fit the experimental results to a numerical model for SLIPS performance within a full-scale AGMD system model. Finally, to evaluate the advantages of using SLIPS within an AGMD system, we compared the performance of SLIPS with filmwise condensation (untreated copper) (Fig. 2d). Additionally, because the AGMD module does not permit in-situ visualization of the condensation, we performed condensation on SLIPS in an open environment to gain a better understanding of the condensation regime.

## Methods

To obtain the experimental data, we used an AGMD test setup with a custom data acquisition and control system (LabView). Computational models of AGMD discretize the membrane module along the length and balance heat and mass transfer interactions, while quantifying concentration and thermal boundary layer effects. Models are adapted from prior work which has been previously published and validated with larger-scale systems^17,19,22^. To determine water droplet sizes on surfaces, we imported high-resolution images of the surfaces into Digimizer, an image analysis software^53^. Within the software, a scale bar in each image was used as a reference for calibration. Following this, we manually measured the diameter of each droplet to accurately quantify the droplet sizes on the surfaces.

### Fabrication of SLIPS

**Fig. 3:**
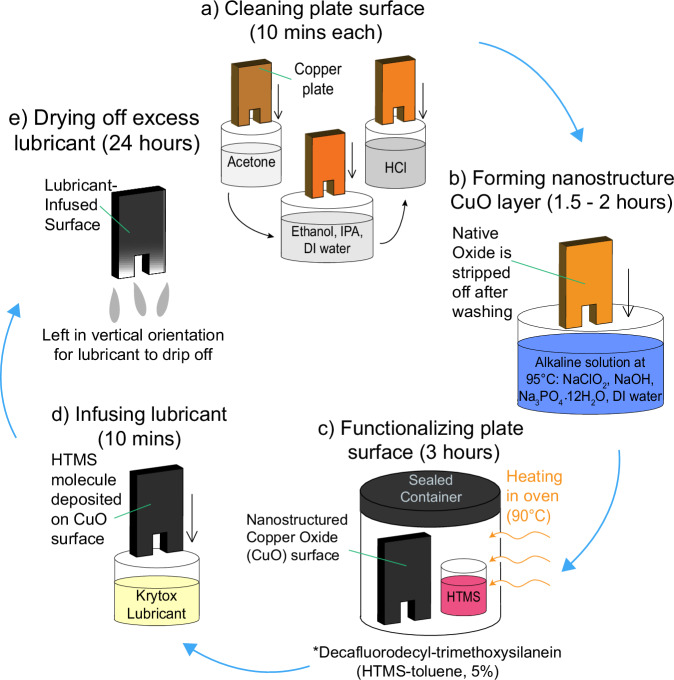
SLIPS fabrication process. **a** The as-obtained Cu surfaces are initially cleaned with acetone, ethanol, isopropanol, and DI water in succession, followed by removal of the native oxide layer in a 2.0 M HCl solution. **b** CuO nanostructures are then grown via chemical oxidation on the cleaned surface by placing the samples in an alkaline solution. **c** The surface is then functionalized by conformally coating (chemical vapor deposition) a silane monolayer. **d** For SLIPS, the functionalized nanostructured surface is dip coated in the lubricant of choice. **e** Excess lubricant is removed via gravity and drying. DI and HTMS are abbreviations of Deionized water and heptadecafluorodecyl-trimethoxysilane, respectively.

SLIPS were fabricated on copper plates with a multistep process that included surface cleaning, removal of the native oxide layer, growing thermally conductive copper oxide nanostructures, functionalization to render the surface hydrophobic, and infusing with a lubricant (Fig. 3). The surface offers a low-thermal-resistance transport pathway by using a thermally conductive substrate (copper), a nanostructured coating with negligible thermal resistance, and an equally thin layer of low surface tension lubricant (Krytox). The lubricant that has been added is held within the surface structures due to capillary forces, which means that the thickness of the coating with and without the lubricant is the same. Previous research on similar surfaces has demonstrated that the additional thickness resulting from the surface structures does not affect the numerical model significantly and does not add any extra thermal resistance^51,54^.

**Fig. 4:**
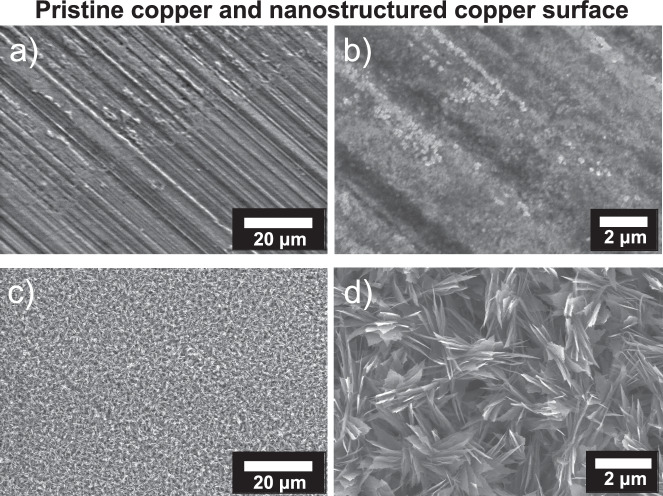
Surface characterization of copper and nanostructured copper oxide. Scanning electron microscopy (SEM) of **a**, **b** pristine copper surface and **c**, **d** nanostructured copper oxide (CuO) surface after surface functionalization with heptadecafluorodecyl-trimethoxysilane (HTMS).

The SLIPS substrates were fabricated similarly to the untreated copper via CNC (Computer Numerical Control) machining. First, the copper surface was cleaned to remove all contaminants followed by the removal of the native oxide layer (Fig. 3a). Cleaning involved dipping the plate in acetone for 10 min followed by rinsing with ethanol, isopropyl alcohol, and deionized water (DI) in succession. The cleaned copper surface was then dipped in a 2 M HCl acid solution for 10 min to remove the native oxide film on the surface. To form a uniform nanostructured copper oxide film, the clean copper surface was held in an alkaline solution of NaClO_2_, NaOH, Na_3_PO_4_.12H_2_O, and DI water (3.75:5:10:100 wt%) maintained at 95 °C for 10 min (Fig. 3b). The oxidation process resulted in a thin ( ~ 300 nm) Cu_2_O layer that further oxidized to form CuO nanostructures ( ~ 1 µm)^27,33^. The nanostructured CuO surface was functionalized using atmospheric pressure chemical vapor deposition (CVD) with heptadecafluorodecyl-trimethoxysilane (HTMS) (Fig. 3c). To do so, the sample was placed in a small glass vial with a 1 mL HTMS-toluene solution (5% v/v), then sealed in a container as shown in Fig. 3c. The sample was then placed in a furnace maintained at 90 °C for 3 h. The low surface energy silane coating rendered the structured copper surface superhydrophobic, resulting in advancing and receding water droplet contact angles of ${\theta }_{a}=161.4\pm 2^{\circ }$, ${\theta }_{r}=156.6\pm 3^{\circ }$, as measured using a goniometer (ramé-hart model 290). The scanning electron microscopy (SEM) images in Fig. 4 show the pristine copper surface and the knife-like CuO nanostructures grown on the copper surface (Fig. 3).

To create the SLIPS, the functionalized nanostructured CuO sample was then dipped into Krytox 16,256, a fluorinated lubricant, for 10 min. Fluorinated oil was chosen as an infusing lubricant because it develops stable and robust SLIPS^47,55^. The lubricant’s low surface tension allows the fluid to wick into the surface nanostructures, while the capillary forces preventing drainage^56^. After dip coating, the samples were left standing vertically for 24 h to drain off the excess oil. The final composition of the SLIPS surface is the lubricant Krytox filled within the functionalized nanostructured CuO surface. Contact angle measurements performed using 5 µl water droplets placed on the fabricated SLIPS show advancing and receding contact angles of ${\theta }_{a}=115.6\pm 1.8^{\circ }$ and ${\theta }_{r}=113.9\pm 2.4^{\circ}$. All condensing surfaces within the AGMD apparatus were approximately 65 mm in height, 38 mm in width and 3.4 mm in thickness.

### Apparatus design

**Fig. 5:**
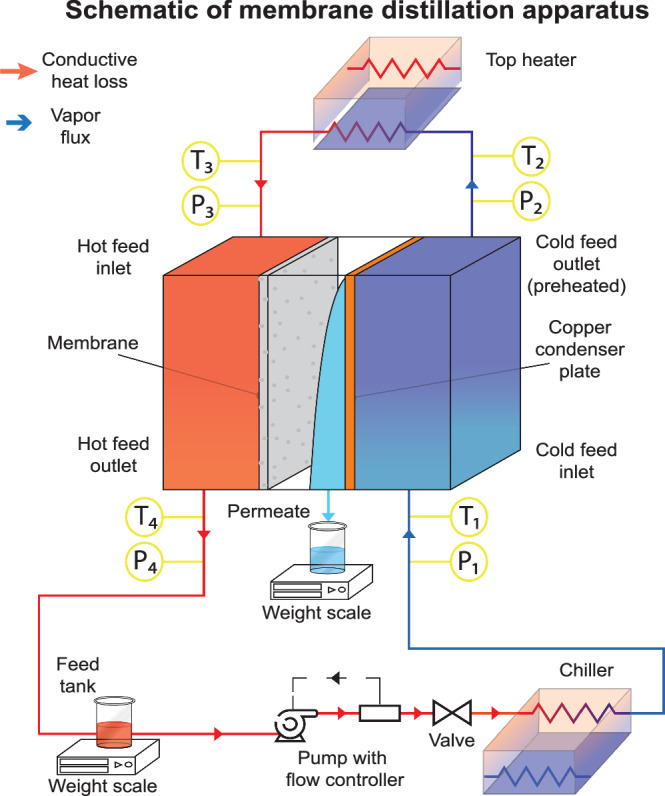
Closed loop countercurrent flow schematic of membrane distillation apparatus. Schematic of MD apparatus and its various components when operating in closed loop, countercurrent flow with flow directions. P and T represent pressure and temperature sensors respectively.

The experimental setup was designed to test a wide variety of MD configurations, with high accuracy measurement of flow rates and temperatures at the inlet and outlet of each channel. This experimental setup consists of two components. The first is the AGMD module (center of Fig. 5) where desalination occurs, and the second is a flow control and measurement system, built by the company Convergence (Figure S1).

As seen in Fig. 5, the desalination process begins as saline feed is pumped from the feed tank (bottom left) through a chiller (bottom right), where it is cooled. The chilled saline feed then enters the cold side of the AGMD module and cools the condensing plate, while simultaneously being preheated by a top heater. The preheated saline feed is further heated by the top heater before entering the hot feed side, where it interacts with the membrane and flows out of the module to the feed tank. Evaporated water from the hot feed side condenses on the condenser plates and is collected as permeate and measured by a weight scale.

The temperatures *T*_*1*_, *T*_*2*_, *T*_*3*_, and *T*_*4*_ are used with the heat capacity rate ṁc_p_ of each stream to calculate the thermal efficiency of the system^26^. This is discussed further in the numerical modeling section. Counterflow of feed and cooling water in the membrane module provides superior heat recovery and is thus nearly universally used in MD^8,47^. The AGMD module was wrapped with two layers of insulation to prevent heat loss. More details about the setup are presented in supplemental sections S1 and S2.

The thickness of the air gap was maintained with plastic mesh spacers. These stiff supports hold up the membrane against hydraulic pressure and are chosen to minimize the influence of membrane warping on condensation. To minimize membrane warping, a coarse mesh and fine mesh were used together. The fine mesh minimizes membrane bulging into the gap. Meanwhile, the course mesh was modified to avoid influence on condensation, usually neglected in MD^18^. Vertical spacer wires in the coarse mesh were removed, so that gaps were 14 mm in length and 3.5 mm in width. The total gap size was 1.54 mm (the width of two spacer wire diameters). However, membrane bulging reduced this slightly, by 0.1–0.3 mm. While the meshes used were non-conducting to minimize their influence, past studies have shown conductive (copper) meshes^18^ can significantly enhance performance. Furthermore, the combination of slippery microchannel/mesh condensers^18^ have shown extremely high heat transfer coefficients and should be explored in MD in future work. Additionally, extremely superhydrophobic surfaces, those inducing jumping droplets showed superior MD performance enhancement^29^, and perhaps could find synergy with SLIPS and microchannel studies.

### Varied condensing surfaces

Two different condensing surfaces, an untreated copper surface, and SLIPS on copper, were fabricated and tested in the system. The AGMD module was operated at hot feed side temperatures of 50 °C, 60 °C, 70 °C, and 80 °C while maintaining a constant temperature difference between the hot and cold side inlets (*∆T* = 20 °C) and a constant flow rate (10 L/h). The feed composition represents salt water (35 g/kg NaCl).

**Fig. 6:**
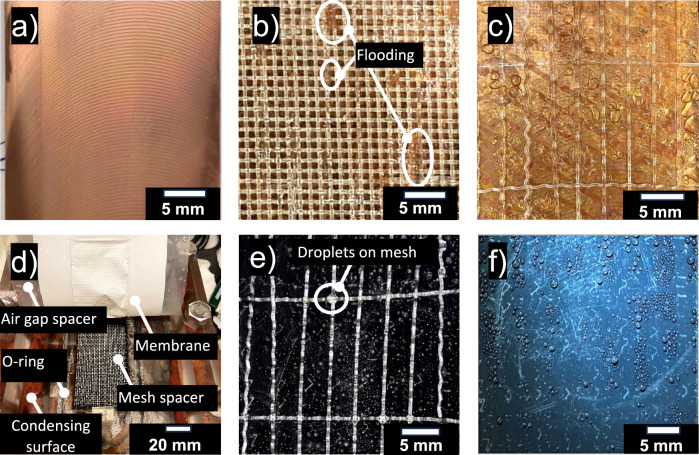
Air gap membrane distillation (AGMD) condensation on untreated copper surface and slippery liquid infused porous surface (SLIPS). **a** Untreated copper template before use in AGMD. **b**) Coarse mesh above the copper plate. Flooding may have occurred at the locations shown with darker patches, where water may have seeped into the mesh. Tested at AGMD tests ${T}_{h,{in}}\,$= 50 °C and ${T}_{c,{in}}\,$= 30 °C. **c** The top section of the untreated surface with large droplets. **d**) SLIPS inside the AGMD module. The AGMD module has been opened to show the placement of the condensing plate and mesh. **e** Dropwise condensation on certain regions of coarse mesh, especially at intersections of threads above the SLIPS surface. Tested at ${T}_{h,{in}}=70$°C and ${T}_{c,{in}}=50$ °C in AGMD. **f** Fine droplet condensation along the length of the SLIPS surface with no signs of filmwise condensation or large droplets.

Each experiment involved setting up the hot feed side temperature and the temperature difference (*∆T)* across the membrane to the previously mentioned temperatures by varying the heater temperature and chiller temperature. Once the temperatures were stabilized the experiment was run for a minimum of 30 min to collect a measurable amount of condensate water. The same process is repeated for the remaining 3 hot inlet temperatures to complete one dataset. The MD system is then stopped, and the AGMD module is carefully separated from the system and placed horizontally. Each layer of the AGMD module is then separated to access the condensing surface as shown in Fig. 6d. The membrane and mesh are removed step by step with the aim of not disturbing the condensation pattern on the condensing surface. The images of condensation are captured using an iPhone 11 camera for measuring and comparing the condensation pattern as shown in Fig.6. This entire process is conducted 3 times for reproducibility.

The water flux of each test was measured by the weight of water condensation in the collector and calculated by

1 $${J}_{{{\rm{p}}}}\left[{{\rm{kg}}}/{{{\rm{m}}}}^{2}{{\rm{h}}}\right]=\frac{\varDelta {\dot{m}}_{{{\rm{p}}}}}{{At}}$$

where ${J}_{{{\rm{p}}}}$ is membrane water flux, kg/m^2^/hour, ${\dot{m}}_{{{\rm{p}}}}$ is the mass flow rate of permeate water, $A$ is the membrane area.

The thermal efficiency (${\eta }_{{{\rm{thermal}}}}$) of the membrane and energy efficiency (gained output ratio (GOR)) of the module were calculated by refs. ^37,58,59^:

2 $${\eta }_{{{\rm{thermal}}}}\left[ \% \right]=100\times \frac{{JA}{h}_{{{\rm{fg}}}}}{{{\dot{m}}_{{{\rm{f}}}}c}_{p,c}({{T}_{{{\rm{c}}},{{\rm{out}}}}-T}_{{{\rm{c}}},{{\rm{in}}}})}$$

3 $${{\rm{GOR}}}={\dot{m}}_{{{\rm{p}}}}\times \frac{{h}_{{{\rm{fg}}}}}{{\dot{m}}_{{{\rm{c}}}}{c}_{p,c}\left({T}_{{{\rm{h}}},{{\rm{in}}}}-{T}_{{{\rm{c}}},{{\rm{out}}}}\right)}$$

where ${h}_{{{\rm{fg}}}}$ is the enthalpy of evaporation, ${c}_{p}$ is heat capacity, ${\dot{m}}_{{{\rm{f}}}}$ is the mass flow rate of feed water, and ${T}_{{{\rm{c}}},{{\rm{out}}}}$, ${T}_{{{\rm{c}}},{{\rm{in}}}}$ and ${T}_{{{\rm{h}}},{{\rm{in}}}}$ are the temperature of outlet and inlet of the cold side and the temperature of inlet of hot side, respectively. Additionally, all abbreviations and symbols are organized in Table 1.

**Table1:** Nomenclature

*Acronyms*	
AGMD	Air gap membrane distillation
SLIPS	Slippery liquid infused porous surfaces
GOR	Gained output ratio
CAH	Contact angle hysteresis
*Roman Symbols*	
$h$	Heat transfer coefficient, W/m^2^K
$k$	Thermal conductivity of water, W/m*K
$g$	Gravitational acceleration, m/s^2^
${h}_{{fg}}$	Latent heat of vaporization, J/kg
${r}_{{{\rm{d}}}}$	Departing droplet radius, m
${r}_{{{\rm{i}}}}$	Effective length equating conduction and Interfacial resistance, m
${r}_{{{\rm{t}}}}$	Minimum droplet radius, m
${R}_{{{\rm{g}}}}$	Gas constant for water vapor J/kg*K
${T}_{{{\rm{sat}}}}$	Saturation temperature, K
$\Delta T$	Difference between surface and saturation Temperature, K
$J$	Vapor flux through the membrane, kg/m^2^h
${M}_{{{\rm{w}}}}$	Molecular weight of water, kg/mol
$C$	Molar concentration, mol/m^3^
${c}_{p}$	Heat capacity, J/kg*K
${d}_{{{\rm{gap}}}}$	Air gap width, m
${D}_{{{\rm{w}}}-{{\rm{a}}}}$	Diffusion coefficient of water in air, m^2^/s
$x$	Mole fraction
${T}_{{{\rm{h}}},{{\rm{in}}}}$	Hot side inlet temperature, K
${T}_{{{\rm{h}}},{{\rm{out}}}}$	Hot side outlet temperature, K
${T}_{{{\rm{c}}},{{\rm{in}}}}$	Cold side inlet temperature, K
${T}_{{{\rm{c}}},{{\rm{out}}}}$	Cold side outlet temperature, K
*Greek symbols*	
$\delta$	Permeate film thickness, m
$\sigma$	Surface tension, kg/s^2^
$\theta$	Contact angle
$\rho$	Liquid water density, kg/m^3^
${\rho }_{{{\rm{v}}}}$	Water vapor density, kg/m^3^
$\pi$	pi
$\gamma$	Heat capacity ratio
*Subscripts*	
$a$	Air gap
$i$	Condensate film interface
$m$	Membrane

### Numerical modeling

A one-dimensional finite–difference-based numerical model was developed to capture the transport phenomena and thermodynamics in AGMD systems. The computational domain of each discretized slice included the hot and cold channels, membrane, and gap. The model was discretized along the length of the module, with properties assumed to be constant across the width, (dimensions mentioned in experimental methodology section and detailed dimensions of system can be found in S1). Transport processes and associated property variations across the depth were quantified using thermal and concentration boundary layers. Additionally, mass and energy conservation equations were formulated for each computational element. Heat and mass transport in the gap were modeled with a growing film thickness for filmwise condensation, and correlations from the literature for dropwise condensation on SLIPS, which were calibrated to experimental results. The resulting set of coupled equations were solved iteratively using Engineering Equation Solver (EES). The solution methodology adopted is based on previous studies by the authors^18,57^ and accurately captures the energy efficiency of MD systems. For brevity, new changes to these models are described in the following sections, while these past studies are recommended for further detail^18,57^.

#### Dropwise condensation

Enhancements in energy efficiency and permeate production using SLIPS were modelled numerically using an AGMD configuration with correlations for dropwise condensation. Dropwise condensation on enhanced surfaces has been examined previously to study the effects of gravity on droplet shedding and condensate removal^59,60^. In this study, we used a correlation developed by Bonner^61^ to calculate the heat transfer coefficient associated with dropwise condensation. Several reviews and studies have used this relation for their analysis to establish credibility^34,62–65^. A heat flux dependent correlation was obtained by fitting our experimental data with a correlation for dropwise condensation.

4 $$h=2.7\frac{k}{{r}_{d}^{1/2}{r}_{i}^{1/4}{r}_{t}^{1/4}}\left(\frac{\sin \theta }{1-\cos \theta }\right)$$

where *k* is the thermal conductivity of water and *θ* the contact angle of water on the condenser surface. This correlation involves several droplet radii to capture the physics where ${r}_{d}$ is the departing droplet radius, ${r}_{i}$ the effective length equating conduction and interfacial resistance, and ${r}_{t}$ the minimum droplet radius. These radii ${r}_{d}$, ${r}_{i}$ and ${r}_{t}$ are given as a function of fluid properties and the temperature difference between the surface and saturation.

5 $${r}_{d}={\left(\frac{\sigma }{\rho g}\right)}^{1/2}$$

6 $${r}_{i}=\frac{k{T}_{{{\rm{sat}}}}}{{\rho }_{v}{h}_{{{\rm{fg}}}}^{2}}\left(\frac{\sin \theta }{1-\cos \theta }\right)\left(\frac{\gamma +1}{\gamma -1}\right){\left(\frac{{R}_{g}{T}_{{{\rm{sat}}}}}{2\pi }\right)}^{1/2}$$

7 $${r}_{t}=\frac{2\sigma {T}_{{{\rm{sat}}}}}{\rho {h}_{{{\rm{fg}}}}\Delta T}$$

where *σ* is the surface tension, *ρ* is the density of water, *g* is the acceleration due to gravity, ${T}_{{{\rm{sat}}}}$ is the saturation temperature of vapor at operating gap pressures, ${\rho }_{v}$ is the density of water vapor, ${h}_{{{\rm{fg}}}}$ is the latent heat, *γ* is the heat capacity ratio, ${R}_{g}$ is the specific ideal gas constant for water vapor and ∆T is the difference between the surface temperature and the saturation temperature. Some scatter was observed in the experimental data when compared with the fitted relation, but an error less than 15%, the reported error of the correlation, was maintained at average operating temperatures of MD systems (50–60 °C).

#### Gap transport

Transport modeling of the air gap, using geometric parameters of the experimental apparatus, was used to characterize the membrane flux, condensation rate, and system energy efficiency. Experimental data for baseline tests with filmwise condensation on a copper plate was obtained using the procedure described further in the Results and Discussion section. These flux and efficiency values serve as foundations for comparing the performance of SLIPS-enabled AGMD systems. They also provide a dataset for fitting several model parameters to the experiments to better represent the observed transport physics.

The mass transfer of vapor in the gap was modelled assuming counter flow diffusion of vapor and air. In the equation for vapor flux ${J}_{m}$ below, ${D}_{w-a}$ was fit to better represent the gap transport.

8 $$\frac{{J}_{m}}{{M}_{w}}=\frac{{C}_{a}{D}_{w-a}}{{d}_{{{\rm{gap}}}}-\delta }{\mathrm{ln}}\left(1+\frac{{x}_{i}-{x}_{a,m}}{{x}_{a,m}-1}\right)$$

${M}_{w}$ is the molecular weight of water, ${C}_{a}$ is the molar concentration of air, ${d}_{{{\rm{gap}}}}$ is the gap size, *δ* the film thickness, and *x* the mole fraction of vapor in the gap. Since mesh spacers take up some room in the gap, and the membrane deforms onto the mesh spacer, slight adjustment in fitting ${d}_{{{\rm{gap}}}}$ to experimental results is necessary. Equivalent gap size corrections were made to all model runs achieving uniformity across the results. The thermal conductivity of the gap was also fit to data, as water and spacers exist in the gap. This correction is especially important in filmwise condensation when water becomes trapped in the mesh spacers.

Once the baseline efficiency and flux values from the numerical model were validated experimentally, the same parameters were used to quantify and compare the values for the SLIPS enabled system.

## Results and discussion

The impact of SLIPS-enhanced condensation is compared to condensation with untreated copper surfaces in a lab-scale membrane distillation apparatus. The lab-scale tests are used to inform system-level impact of SLIPS in AGMD and validate numerical models. Validated models are then used to predict the impact of such surfaces in practical-scale systems. The condensation is visualized on SLIPS in an open environment for further concept validation.

### Experimental condenser surface comparison

Filmwise condensation on the untreated copper surface was characterized under varied dimensions, temperature- and flow rate conditions representative of a full-scale AGMD system. Figure 6 shows the untreated copper plate and SLIPS, both before and after testing in the AGMD module. Figure 6a shows the untreated copper plate before it was tested. After testing, flooding can be seen in certain locations of the mesh highlighted in Fig. 6b where the spacer appears darker, or the menisci are visible. Figure 6c shows large, condensed droplets which flood the air gap at their respective locations. It should be noted that even at low temperatures, a typical air gap (1.5 mm in thickness) is prone to flooding.

**Fig. 7:**
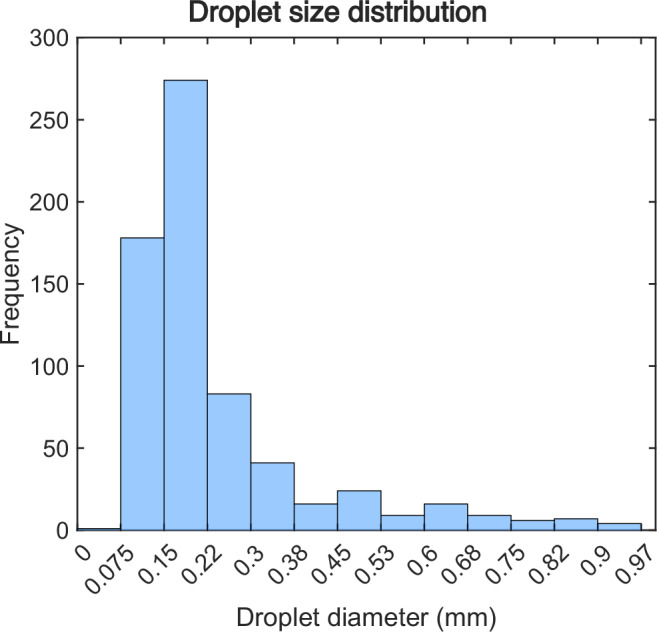
Droplet size distribution of condensed water vapor on a slippery liquid-infused porous surface (SLIPS). Droplet size distribution during dropwise condensation on SLIPS surface, showcasing the improved condensation efficiency attributed to smaller droplets and higher density.

Similarly, the SLIPS substrate was run for AGMD, with gap sizes and gap spacers representative of real AGMD systems. Figure 6 shows the SLIPS before and after running the test in AGMD module. Figure 6d shows how the membrane, mesh spacer, air gap spacer, and condensing surface were placed in relation to one another. For the SLIPS surface, there were fewer visible indications of flooding on the support meshes, which were stacked upon each other. The top mesh was removed to inspect for flooding on the condensing surface (Fig. 6e), which was not present. However, some small condensation droplets were visible on the coarse mesh (Fig. 6e), but their sizes were small compared to the condensation droplets on untreated copper surface (Fig. 6c). After the removal of the coarse mesh from the top of the surface, a clear condensation pattern was visible on the SLIPS, as illustrated in Fig. 6f. To investigate the droplet diameters on SLIPS, image analysis software was utilized to examine this figure, an approach not feasible for the untreated copper surface due to the absence of distinct droplets. Figure 7 displays the distribution of droplets on SLIPS, with the analysis revealing that the average diameter of these condensed droplets was approximately 0.24 mm. Compared to the droplets present on the untreated copper surface, the droplets on SLIPS were notably smaller and more densely distributed. Owing to the low contact angle hysteresis (CAH < 2), droplets can readily form and glide over the lubricant layer, facilitating coalescence and removal from the surface, thereby creating new nucleation sites. This phenomenon improves the mobility of droplets and augments dropwise condensation efficiency. Consequently, no signs of film formation or flooding are observed on the modified surface.

### System-level performance impact of SLIPS in AGMD

**Fig. 8:**
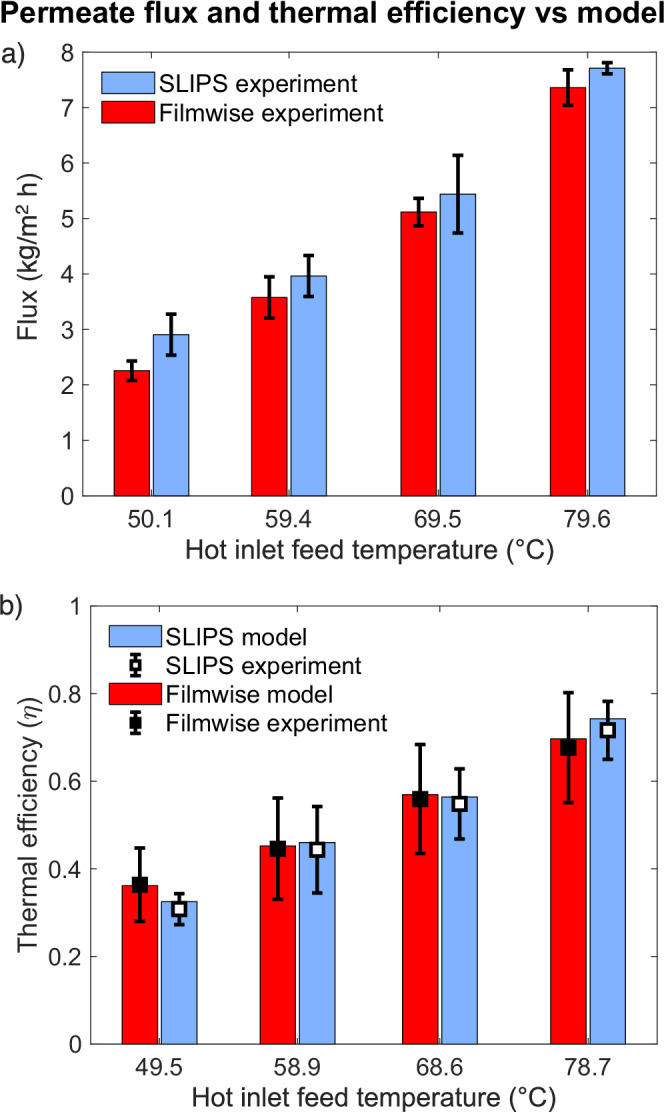
Comparison of membrane flux and thermal efficiency in slippery liquid infused porous surface (SLIPS) vs. untreated copper surfaces. **a** Plot of membrane flux versus hot inlet temperature (°C), comparing the SLIPS and untreated copper surface. **b** Comparison of experimental and model thermal efficiency for untreated copper surface and SLIPS. Tests were conducted with an air gap of 1.54 mm, ∆T = 20 °C between hot and cold inlets, flow rate of 10 L/h, and a feed salinity of 35 g/kg. Each test was performed three times at each temperature, and the results include the average and standard deviation.

To evaluate the effects of SLIPS condensation on thermal efficiency at practical system scales, the flux and temperatures were measured and used as inputs to the model. Notably, for these experiments at small sizes, measured system energy efficiency metrics like the GOR do not provide relevant insights. Figure 8 compares the permeate flux production and thermal efficiency of SLIPS and the untreated surface using both experimental data and model predictions. Figure 8a reveals that, throughout the temperature range, the flux produced by SLIPS consistently surpasses that of untreated copper. The pure water flux also increases rapidly with temperature as vapor pressure is an exponential function of temperature^66,67^. The water flux increased in SLIPS likely duo to a reduced mass transport resistance from SLIPS condensation, which provides many nucleation sites, good droplet shedding, and reduced condensate film thickness^68,69^.

**Fig. 9:**
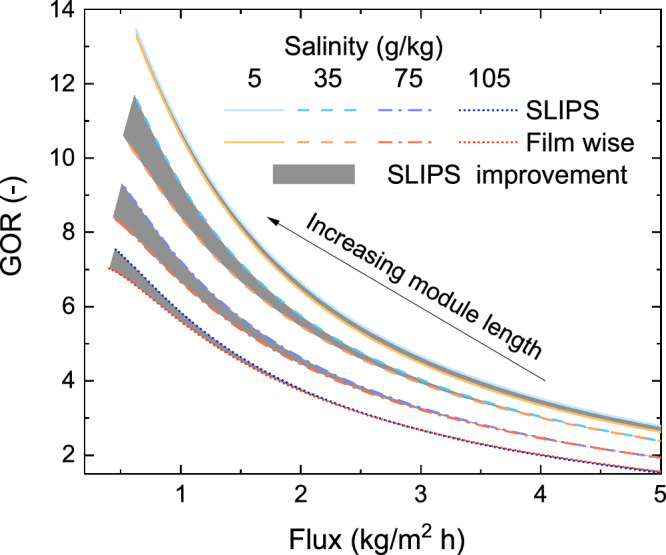
SLIPS performance improvement at scale. Energy efficiency (GOR) and permeate production for slippery liquid infused porous surface (SLIPS) -adjusted models and a filmwise condensing regime are shown at various salinities. Efficiency curves are plotted at the optimal gap size at each salinity for SLIPS. For filmwise condensation, the efficiency frontiers at 5 and 35 $\frac{g}{{kg}}$ salinities have been plotted at the gap sizes that prevent flooding (0.1 and 0.14 mm respectively) whereas for other salinities gap size is identical to SLIPS. Module length is varied from 0.5 to 30 m to properly capture the GOR value peaks for the considered salinity range of 5–105 $\frac{g}{{kg}}$. The shaded areas indicate the improvements made through SLIPS.

Thermal efficiency considers the local heat transfer, as a rate of that from condensation and the total heat transfer^70^. Conduction losses to the environment and across the air gap increased for SLIPS surface, resulting in thermal efficiency being very similar. The 3- dimensional geometry of droplets reduced the distances for conduction losses through the air, and the apparatus was too small for flooding effects of conventional surfaces to develop a thick condensate film. However, as increased flux provides more water for the same energy input, we will see an improvement of the GOR-Flux frontier (Fig. 9) when only flux improves.

In addition, Fig. 8b highlights the strong agreement between the model and experiments in predicting the thermal efficiency increase with temperature. This is expected, as evaporation rate increases exponentially at higher temperatures, becoming relatively large compared to thermal losses. Full-scale systems typically exhibit lower thermal losses, resulting in higher efficiencies than the experimental results shown in this study. Water entrapment in the mesh spacer may alter the effective gap conductivity and thermal resistances, which may explain the slight deviation between experiment and model results. These findings serve as inputs to a model examining efficiency trade-offs in MD, and energy efficiency (GOR) versus permeate flux over a wide range of salinities.

### Practical scale performance

Demonstrating GOR vs Flux will require the development of a full-scale membrane module, and thus it is standard to use modeling to predict the performance of larger systems^22,71,72^. The energy efficiency (GOR) and average flux of small-scale tests and full-scale systems may vary significantly. The air gap depth significantly affects the system performance, and the optimal depth varies with membrane area, module length, and feed salinity. Notably, when considering high salinities, AGMD is the most cost competitive and efficient compared to other MD configurations like PGMD and CGMD. For each condition, the air gap depth is defined as the depth that maximizes efficiency (GOR) without flooding. The GOR-flux tradeoff gives a direct comparison for efficiency improvements from enhanced condensation via SLIPS in AGMD. The performance of AGMD systems using SLIPS and filmwise condensation was modelled using experimentally validated parameters and compared at different module lengths and feed inlet salinity as shown in Fig. 9. At smaller module lengths that correspond to lab scale tests, the energy efficiency (GOR) is low and the permeate production per unit area (flux) is high nearly. In comparison, SLIPS demonstrated enhanced performance over filmwise condensation at high salinities, attributed to higher GOR at increased membrane areas with lower flux, as depicted in Fig. 9. Conversely, at lower salinities and greater areas with higher permeate flux, the two systems’ performance converged, as shown in Fig. 9, far right. The advantage is largely due to avoiding flooding, which is more likely at large system sizes and high salinities. Notably, when considering high salinities, AGMD is more cost competitive and relatively efficient compared to other MD configurations like PGMD and CGMD.

**Fig. 10:**
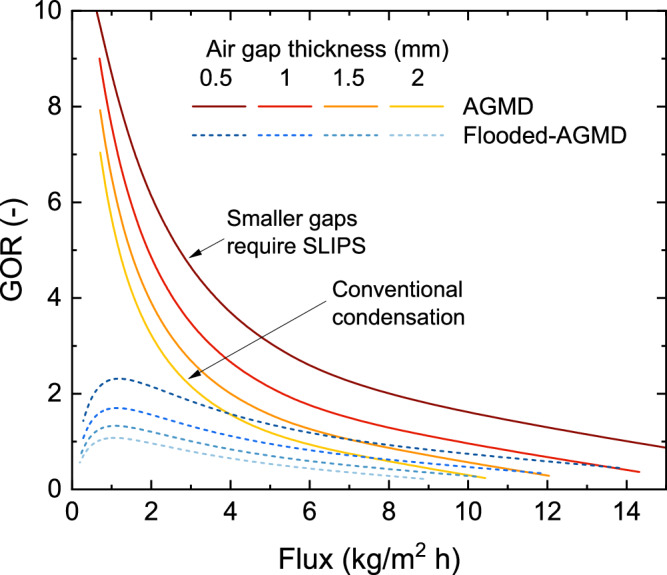
Effect of flooding of air gap in air gap membrane distillation (AGMD) performance. Energy efficiency (GOR) vs Flux for different gap sizes in AGMD and Flooded-AGMD. Flooded-AGMD refers to an AGMD setup where the gap is flooded with water permeate, also known as PGMD in the literature^18,77^. Each plot created with module length varies from 0.5 to 30 m, with a salinity of 105 g/kg. Based on previous works^67,78–83^, the flux of commercial modules operates <6 kg/m^2^h in high salinity.

To demonstrate the impact of flooding or the water bridge on the performance of the AGMD module, a comparison was made between AGMD and Flooded-AGMD (PGMD) (Fig. 10). The results indicate that when the gap is flooded with permeate, the performance of AGMD decreases due to increased heat conduction loss from water bridging in the air gap^23^. For example, the GOR reduced by 85% when the 2 mm gap was flooded with permeate in the high-length module. The capillary length of water is 2.7 mm^73^, and on typical metallic surfaces, most droplets will not descend until they approach that length, so many AGMD modules use gaps of 2 mm or larger^74–76^. In contrast, the SLIPS droplet diameters depart well below 0.5 mm, as shown in Fig. 7, enabling performance enhancements that exceed 40%. Therefore, the primary advantage of this approach is better droplet shedding, rather than changes in the mass transfer coefficient. SLIPS reduced droplet adhesion and significantly facilitated droplet shedding, providing an alternative approach to maintain performance in large-scale modules while eliminating air gap flooding. The improved droplet mobility does increase mass transfer, but this effect is far smaller than the advantage of decreasing gap thickness.

## Conclusions

Through rigorous experimental and computational analysis, this study is the first to consider the application of SLIPS condensation to not only AGMD but to any desalination technology. The experimental performance from SLIPS was compared to an untreated copper surface. Several key conclusions were drawn:

- SLIPS showed formation of smaller size condensate droplets, which enabled the practical operation of AGMD at smaller air gap thicknesses by avoiding flooding. According to the models, the efficiency improvements gained through smaller air gaps is the most significant benefit from the use of SLIPS.
- The SLIPS condenser showed higher permeate production at all temperatures, compared to the untreated copper condenser. This enhancement can be attributed to faster droplet shedding, which reduces the mass transfer resistance by increasing the available area for droplet nucleation.
- Model fitting by matching transport resistances provides accurate prediction of thermal efficiency and flux for SLIPS and untreated copper condensing surfaces. The models show a better fit at higher permeate fluxes.
- Membrane support meshes, which are usually neglected in AGMD models, do influence performance by trapping droplets and may explain small deviations between observed data and model predictions.
- The energy-optimal air gap depth is different for SLIPS and untreated copper condensing surfaces. The optimal depth varies by salinity and flux.
- Performance differences between SLIPS and untreated copper surfaces are most significant in high-flooding conditions (large areas, low salinities), as air gap sizes for baseline filmwise condensation must be larger to avoid flooding.

Further investigation should consider the impact of lubricant leakage on the permeate condensate quality. Additionally, the choice and design of lubricants may be further analyzed for stability, toxicity, and separability. Moreover, as long as liquid lubricant is used, the durability of SLIPS is not as good as the newly developed quasi-liquid surface. Therefore, potential future work may involve exploring hydrophilic SLIPS or quasi-liquid surfaces based on new studies.

## Supplementary information

Supplementary Information

## Data Availability

All relevant data are available upon request from the corresponding author.

## Code availability

Code is available from the corresponding author upon reasonable request.

## References

[1] UNESCO, United Nations World Water Development Report 2020; Water and Climate Change, in: UN-Water, Paris, 2020. 10.1002/9781118786352.wbieg0793.pub2.

[2] Hoekstra, A. Y., Mekonnen, M. M., Chapagain, A. K., Mathews, R. E. & Richter, B. D. Global Monthly Water Scarcity: Blue Water Footprints versus Blue Water Availability. *PLoS One* **7**, e32688 (2012). 22393438 10.1371/journal.pone.0032688 PMC3290560

[3] Gingerich, D. B. & Mauter, M. S. Quantity, Quality, and Availability of Waste Heat from United States Thermal Power Generation. *Environ Sci Technol* **49**, 8297–8306 (2015). 26061407 10.1021/es5060989

[4] Warsinger, D. M., Mistry, K. H., Nayar, K. G., Chung, H. Won & Lienhard, J. H. V, Entropy Generation of Desalination Powered by Variable Temperature Waste Heat. *Entropy* **17**, 7530–7566 (2015).

[5] Lawson, K. W. & Lloyd, D. R. Membrane distillation. *J Memb Sci* **124**, 1–25 (1997).

[6] Alklaibi, A. M. & Lior, N. Membrane-distillation desalination: Status and potential. *Desalination* **171**, 111–131 (2005).

[7] El-Bourawi, M. S., Ding, Z., Ma, R. & Khayet, M. A framework for better understanding membrane distillation separation process. *J Memb Sci* **285**, 4–29 (2006).

[8] Alsaadi, A. S. et al. Modeling of air-gap membrane distillation process: A theoretical and experimental study. *J Memb Sci* **445**, 53–65 (2013).

[9] Hitsov, I., Maere, T., De Sitter, K., Dotremont, C. & Nopens, I. Modelling approaches in membrane distillation: A critical review. *Sep Purif Technol* **142**, 48–64 (2015).

[10] Liu, G. L., Zhu, C., Cheung, C. S. & Leung, C. W. Theoretical and experimental studies on air gap membrane distillation. *Heat and Mass Transfer/Waerme- Und Stoffuebertragung* **34**, 329–335 (1998).

[11] Zarei, M. et al. Extreme Cooling Via Sweeping Gas Membrane Distillation. *Proc. Int. Heat Transfer Conference* **17**, 12 (2023).

[12] Gao, L., Zhang, J., Gray, S. & De Li, J. Modelling mass and heat transfers of Permeate Gap Membrane Distillation using hollow fibre membrane. *Desalination* **467**, 196–209 (2019).

[13] Schwantes, R. et al. Membrane distillation: Solar and waste heat driven demonstration plants for desalination. *Desalination* **323**, 93–106 (2013).

[14] Gude, V. G. Geothermal source potential for water desalination - Current status and future perspective. *Renewable and Sustainable Energy Reviews* **57**, 1038–1065 (2016).

[15] Summers, E. K. & Lienhard, J. H. Experimental study of thermal performance in air gap membrane distillation systems, including the direct solar heating of membranes. *Desalination* **330**, 100–111 (2013).

[16] Guillén-Burrieza, E. et al. Experimental analysis of an air gap membrane distillation solar desalination pilot system. *J Memb Sci* **379**, 386–396 (2011).

[17] Parmar, H. B. et al. Nanofluids improve energy efficiency of membrane distillation. *Nano Energy* **88**, 106235 (2021).

[18] Swaminathan, J. et al. Energy efficiency of permeate gap and novel conductive gap membrane distillation. *J Memb Sci* **502**, 171–178 (2016).

[19] Deshmukh, A. et al. Membrane distillation at the water-energy nexus: limits, opportunities, and challenges. **11** 1177–1196 (2018).

[20] Rezaei, M., Alsaati, A., Warsinger, D. M., Hell, F. & Samhaber, W. M. Long-Running Comparison of Feed-Water Scaling in Membrane Distillation. *Membranes (Basel)* **10**, 1–21 (2020). 10.3390/membranes10080173 PMC7463528 32751820

[21] Tow, E. W. et al. Comparison of fouling propensity between reverse osmosis, forward osmosis, and membrane distillation. *J Memb Sci* **556**, 352–364 (2018).

[22] Swaminathan, J., Chung, H. W., Warsinger, D. M. & Lienhard V, J. H. Energy efficiency of membrane distillation up to high salinity: Evaluating critical system size and optimal membrane thickness. *Appl Energy* **211**, 715–734 (2018).

[23] Warsinger, D. M., Swaminathan, J. & Morales, L. L. J.H.L. V, Comprehensive condensation flow regimes in air gap membrane distillation: Visualization and energy efficiency. *J Memb Sci* **555**, 517–528 (2018).

[24] Khayet, M. & Cojocaru, C. Air gap membrane distillation: Desalination, modeling and optimization. *Desalination* **287**, 138–145 (2012).

[25] Hitsov, I., De Sitter, K., Dotremont, C., Cauwenberg, P. & Nopens, I. Full-scale validated Air Gap Membrane Distillation (AGMD) model without calibration parameters. *J Memb Sci* **533**, 309–320 (2017).

[26] Warsinger, D. M., Nejati, S. & Juybari, H. F. Energy Efficiency Metrics in Membrane Distillation. *Materials and Energy* **17**, 263–288 (2021).

[27] Ji, X., Zhou, D., Dai, C. & Xu, J. Dropwise condensation heat transfer on superhydrophilic-hydrophobic network hybrid surface. *Int J Heat Mass Transf* **132**, 52–67 (2019).

[28] Yan, X. et al. Droplet Jumping: Effects of Droplet Size, Surface Structure, Pinning, and Liquid Properties, ACS Nano 13 (2019) 1309–1323. 10.1021/acsnano.8b06677 30624899

[29] Warsinger, D. E. M. M., Swaminathan, J., Maswadeh, L. A., Lienhard, J. H. & Lienhard V, J. H. Superhydrophobic condenser surfaces for air gap membrane distillation. *J Memb Sci* **492**, 578–587 (2015).

[30] Rose, J. W. Dropwise condensation theory and experiment: a review. *Proc. Institution of Mechanical Engineers, Part A: Journal of Power and Energy*, Vol. 216, 115–128 (2002).

[31] Rose, J. W. Condensation heat transfer fundamentals. *Chemical Engineering Research and Design* **76**, 143–152 (1998).

[32] Collier J.G. & Thome J. R., Convective boiling and condensation., Clarendon Press., 1994.

[33] Enright, R., Miljkovic, N., Alvarado, J. L., Kim, K. & Rose, J. W. Dropwise condensation on micro-and nanostructured surfaces. *Nanoscale and Microscale Thermophysical Engineering* **18**, 223–250 (2014).

[34] Zhang, P., Maeda, Y., Lv, F., Takata, Y. & Orejon, D. Enhanced Coalescence-Induced Droplet-Jumping on Nanostructured Superhydrophobic Surfaces in the Absence of Microstructures. *ACS Appl Mater Interfaces* **9**, 35391–35403 (2017). 28925681 10.1021/acsami.7b09681

[35] Ma, J., Sett, S., Cha, H., Yan, X. & Miljkovic, N. Recent developments, challenges, and pathways to stable dropwise condensation: A perspective. *Appl Phys Lett* **116**, 260501 (2020).

[36] Warsinger, D. M., Swaminathan, J., Morales, L. L. & Lienhard V, J. H. Comprehensive condensation flow regimes in air gap membrane distillation: Visualization and energy efficiency. *J Memb Sci* **555**, 517–528 (2018).

[37] Fattahi Juybari, H., Parmar, H. B., Alshubbar, A. D., Young, K. L. & Warsinger, D. M. Porous condensers can double the efficiency of membrane distillation. *Desalination* **545**, 116129 (2023).

[38] Miljkovic, N. et al. Jumping-droplet-enhanced condensation on scalable superhydrophobic nanostructured surfaces. *Nano Lett* **13**, 179–187 (2013). 23190055 10.1021/nl303835d

[39] Warsinger, D. M., Swaminathan, J., Morales, L. L., Bertoni, M. & Lienhard, J. H. Visualization of droplet condensation in membrane distillation desalination with surface modification: hydrophilicity, hydrophobicity, and wicking spacers, 2017. https://doi.org/http://hdl.handle.net/1721.1/112109.

[40] Li, J., Ueda, E., Paulssen, D. & Levkin, P. A. Slippery Lubricant-Infused Surfaces: Properties and Emerging Applications. *Adv Funct Mater* **29**, 1–13 (2019).

[41] Guo, Z., Monga, D., Shan, L., Boylan, D. & Dai, X. Coarsening-induced disappearing droplets contribute to condensation. *Droplet* **1**, 170–181 (2022).

[42] Guo, Z., Boylan, D., Shan, L. & Dai, X. Hydrophilic reentrant SLIPS enabled flow separation for rapid water harvesting. *Proc. Natl. Acad. Sci. USA* **119**, e2209662119 (2022). 10.1073/pnas.2209662119 PMC9457051 36037348

[43] Guo, Z., Boylan, D., Li, S. & Dai, X. Hydrophilic reentrant SLIPS enabled flow separation for rapid water harvesting. **119**, e2209662119 (2022). 10.1073/pnas.2209662119 PMC9457051 36037348

[44] Anand, S., Paxson, A. T., Dhiman, R., Smith, J. D. & Varanasi, K. K. Enhanced Condensation on Lubricant-Impregnated Nanotextured Surfaces. *ACS Nano* **6**, 10122–10129 (2012). 23030619 10.1021/nn303867y

[45] Park, K. C. et al. Condensation on slippery asymmetric bumps. *Nature 2016* **531**, 78–82 (2016). 7592 531. 10.1038/nature16956 26909575

[46] Monga, D. et al. Quasi-Liquid Surfaces for Sustainable High-Performance Steam Condensation. *ACS Appl Mater Interfaces* **14**, 13932–13941 (2022). 35287435 10.1021/acsami.2c00401

[47] Sett, S. et al. Stable Dropwise Condensation of Ethanol and Hexane on Rationally Designed Ultrascalable Nanostructured Lubricant-Infused Surfaces. *Nano Lett* **19**, 5287–5296 (2019). 31328924 10.1021/acs.nanolett.9b01754

[48] Tsuchiya, H. et al. Liquid-Infused Smooth Surface for Improved Condensation Heat Transfer. *Langmuir* **33**, 8950–8960 (2017). 28826213 10.1021/acs.langmuir.7b01991

[49] Solomon, B. R. et al. Lubricant-Impregnated Surfaces. In *Non-Wettable Surfaces: Theory, Preparation, and Applications*. (eds Ras, R. H. A. & Marmur, A.), 285–318 (The Royal Society of Chemistry, 2016). 10.1039/9781782623953-00285

[50] Xiao, R., Miljkovic, N., Enright, R. & Wang, E. N. Immersion Condensation on Oil-Infused Heterogeneous Surfaces for Enhanced Heat Transfer. *Sci Rep* **3**, 1988 (2013). 23759735 10.1038/srep01988 PMC3680863

[51] Ho, J. Y., Rabbi, K. F., Sett, S., Wong, T. N. & Miljkovic, N. Dropwise condensation of low surface tension fluids on lubricant-infused surfaces: Droplet size distribution and heat transfer. *Int J Heat Mass Transf* **172**, 121149 (2021).

[52] Sett, S., Yan, X., Barac, G., Bolton, L. W. & Miljkovic, N. Lubricant-Infused Surfaces for Low-Surface-Tension Fluids: Promise versus Reality. *ACS Appl Mater Interfaces* **9**, 36400–36408 (2017). 28950702 10.1021/acsami.7b10756

[53] Digimizer Image Analysis Software, (n.d.). https://www.digimizer.com/.

[54] Maeda, Y., Lv, F., Zhang, P., Takata, Y. & Orejon, D. Condensate droplet size distribution and heat transfer on hierarchical slippery lubricant infused porous surfaces. *Appl Therm Eng* **176**, 115386 (2020).

[55] Jahidul Hoque, M. et al. Life Span of Slippery Lubricant Infused Surfaces. *ACS Applied Materials & Interfaces* **14**, 4598–4611 (2022). 35018774 10.1021/acsami.1c17010

[56] Sett, S. et al. Lubricant-Infused Surfaces for Low-Surface-Tension Fluids: The Extent of Lubricant Miscibility. *ACS Applied Materials & Interfaces* **13**, 23121–23133 (2021). 33949848 10.1021/acsami.1c02716

[57] Juybari, H. F., Karimi, M., Srivastava, R., Swaminathan, J. & Warsinger, D. M. Superhydrophobic composite asymmetric electrospun membrane for sustainable vacuum assisted air gap membrane distillation. *Desalination* **553**, 116411 (2023).

[58] Juybari H.F. et al. Performance of membrane distillation technologies, World Scientific Reference Of Water Science 223–266 (2022).

[59] Summers, E. K., Arafat, H. A. & Lienhard, J. H. V, Energy efficiency comparison of single-stage membrane distillation (MD) desalination cycles in different configurations. *Desalination* **290**, 54–66 (2012).

[60] Boreyko, J. B., Zhao, Y. & Chen, C. H. Planar jumping-drop thermal diodes. *Appl Phys Lett* **99**, 1–4 (2011).

[61] Daniel, S., Chaudhury, M. K. & Chen, J. C. Fast drop movements resulting from the phase change on a gradient surface. *Science* **291**, 633–636 (2001). 11158672 10.1126/science.291.5504.633

[62] Bonner, R. W. Correlation for dropwise condensation heat transfer: Water, organic fluids, and inclination. *Int J Heat Mass Transf* **61**, 245–253 (2013).

[63] Ghosh, A., Beaini, S., Zhang, B. J., Ganguly, R. & Megaridis, C. M. Enhancing dropwise condensation through bioinspired wettability patterning. *Langmuir* **30**, 13103–13115 (2014). 25295388 10.1021/la5028866

[64] Kim, D. E., Ahn, H. S. & Kwon, T. S. Experimental investigation of filmwise and dropwise condensation inside transparent circular tubes. *Appl Therm Eng* **110**, 412–423 (2017).

[65] Attinger, D. et al. Megaridis, Surface engineering for phase change heat transfer: A review. *MRS Energy & Sustainability* **1**, 1–40 (2014).

[66] Luo, A. & Lior, N. Study of advancement to higher temperature membrane distillation. *Desalination* **419**, 88–100 (2017).

[67] Eykens, L. et al. Direct contact and air gap membrane distillation: Differences and similarities between lab and pilot scale. *Desalination* **422**, 91–100 (2017).

[68] Yao, W., Wu, L., Sun, L., Jiang, B. & Pan, F. Recent developments in slippery liquid-infused porous surface. *Prog. Org. Coat.* **166**, 106806 (2022).

[69] Gulfam, R., en Huang, T., Lv, C., Orejon, D. & Zhang, P. Condensation heat transfer on phase change slippery liquid-infused porous surfaces. *IJHMT* **185**, 122384 (2022).

[70] Juybari, H. F. et al. Unifying Efficiency Metrics for Solar Evaporation and Thermal Desalination. *ACS Energy Lett.* **9**, 4959–4975 (2024).

[71] Swaminathan, J., Chung, H. W., Warsinger, D. M. & Lienhard V, J. H. Membrane distillation model based on heat exchanger theory and configuration comparison. *Appl Energy* **184**, 491–505 (2016).

[72] Lin, B. R. et al. Comparative Energetics of Various Membrane Distillation Configurations and Guidelines for Design and Operation. *Membranes 2023* **13**, 273 13 (2023). VolPage273. 10.3390/membranes13030273 PMC10056151 36984660

[73] Adera, S. et al. Enhanced condensation heat transfer using porous silica inverse opal coatings on copper tubes. *Sci. Rep.* **11**, 10675 (2021). 10.1038/s41598-021-90015-x PMC8140112 34021211

[74] Zhang, Y. & Guo, F. Mitigating near-surface polarizations in membrane distillation via membrane surface decoration. *Desalination* **579**, 117507 (2024).

[75] Khalifa, A., Lawal, D., Antar, M. & Khayet, M. Experimental and theoretical investigation on water desalination using air gap membrane distillation. *Desalination* **376**, 94–108 (2015).

[76] Gopi, G. et al. Performance, energy and economic investigation of airgap membrane distillation system: An experimental and numerical investigation. *Desalination* **551**, 116400 (2023).

[77] Ali, A. et al. Progress in module design for membrane distillation. *Desalination* **581**, 117584 (2024).

[78] Bindels, M. & Nelemans, B. Theoretical analysis of heat pump assisted air gap membrane distillation. *Desalination* **518**, 115282 (2021).

[79] Zaragoza, G., Ruiz-Aguirre, A. & Guillén-Burrieza, E. Efficiency in the use of solar thermal energy of small membrane desalination systems for decentralized water production. *Appl Energy* **130**, 491–499 (2014).

[80] Andrés-Mañas, J. A., Ruiz-Aguirre, A., Acién, F. G. & Zaragoza, G. Performance increase of membrane distillation pilot scale modules operating in vacuum-enhanced air-gap configuration. *Desalination* **475**, 114202 (2020).

[81] Winter, D. et al. Comparative analysis of full-scale membrane distillation contactors - methods and modules. *J Memb Sci* **524**, 758–771 (2017).

[82] Winter, D., Koschikowski, J. & Ripperger, S. Desalination using membrane distillation: Flux enhancement by feed water deaeration on spiral-wound modules. *J. Memb. Sci.* **423**, 215–224 (2012).

[83] Adham, S., Minier-Matar, J. & Hussain, A. Pilot plant evaluation of membrane distillation for desalination of high-salinity brines. *Appl Water Sci* **13**, 1–10 (2023).

