# Supplementary material for: Slippery liquid infused porous surface (SLIPS) condensers for high efficiency air gap membrane distillation
Source: Commun Eng. 2025 Mar 15;4:48. doi: 10.1038/s44172-025-00348-y (PMC11910583; doi:10.1038/s44172-025-00348-y)
Supplement: Supplementary file 1 — Supplementary Information [file 44172_2025_348_MOESM1_ESM.pdf]

Slippery Liquid Infused Porous Surface (SLIPS) Condensers for High Efficiency Air Gap Membrane Distillation

Yashwant S Yogi[1], Harsharaj B Parmar[1], Hamid Fattahi Juybari[1], Sina Nejati[1], Akshay K Rao[1], Rishav Roy[1], Mojtaba Zarei[1], Longnan Li[2], Soumyadip Sett[2], Abhimanyu Das[1], Nenad Miljkovic[2,3,4,5], Justin A Weibel[1], David M Warsinger[1*]

[1] School of Mechanical Engineering and Birck Nanotechnology Center, Purdue University, West Lafayette, Indiana 47907, USA

[2] Department of Mechanical Science and Engineering, University of Illinois, Urbana, Illinois 61801, United States

[3] Materials Research Laboratory, University of Illinois, Urbana, IL, 61801, USA

[4] Department of Electrical and Computer Engineering, University of Illinois, Urbana, IL, 61801, USA

[5] International Institute for Carbon Neutral Energy Research (WPI-I2CNER), Kyushu University, 744 Motooka, Nishi-ku, Fukuoka 819-0395, Japan

* Corresponding Author: (D.W.), Email: dwarsing@purdue.edu

1. Supplemental Materials

S1: Apparatus flow control and data acquisition system

S2: Membrane module exploded view

S3: Correlation validation

S4: SLIPS condensation visualization

S5: Practical energy efficiency enhancements using SLIPS

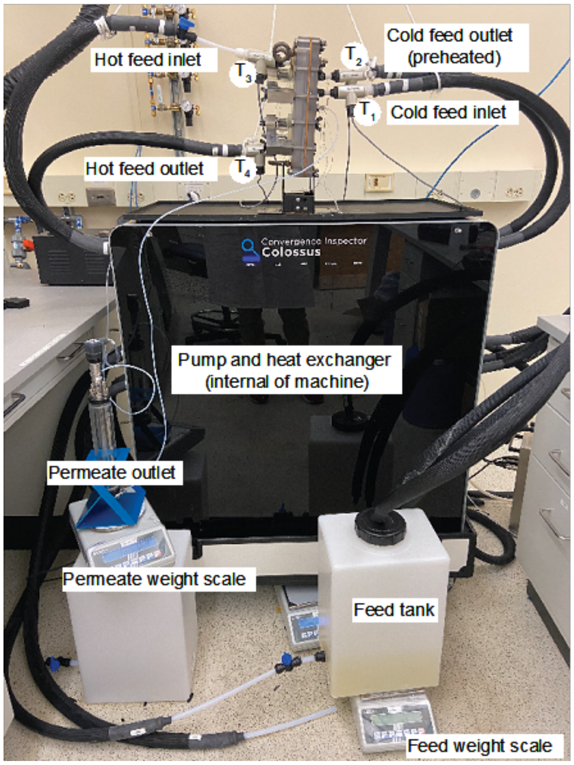

Figure S1: Apparatus. Laboratory scale MD photo of the apparatus used in this study.

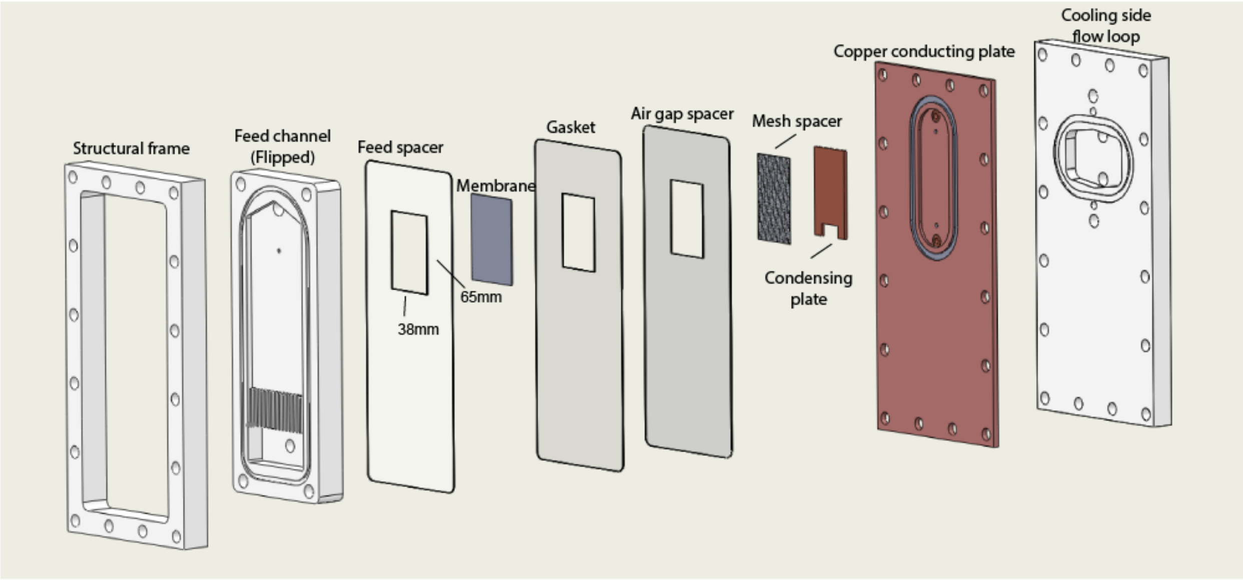

Figure S2: Membrane module exploded view. CNC polycarbonate plates (left and far right) provide channels for fluid flow. Spacer plates provide for an adequate air gap depth. A condensing plate separates the condensate from the cooling/preheating channel. A spacer and gasket around the membrane achieve a good seal, while O-rings achieve seals elsewhere. The membrane module design considers representative channel depths, gap depths, flowrates, and operating parameters that are similar to a full-scale system [1,2]. The channel length and surface area must also be sufficient to have measurable temperature drops to calculate the thermal efficiency. Insulating plastic materials are chosen to minimize adverse temperature gradients and heat losses.

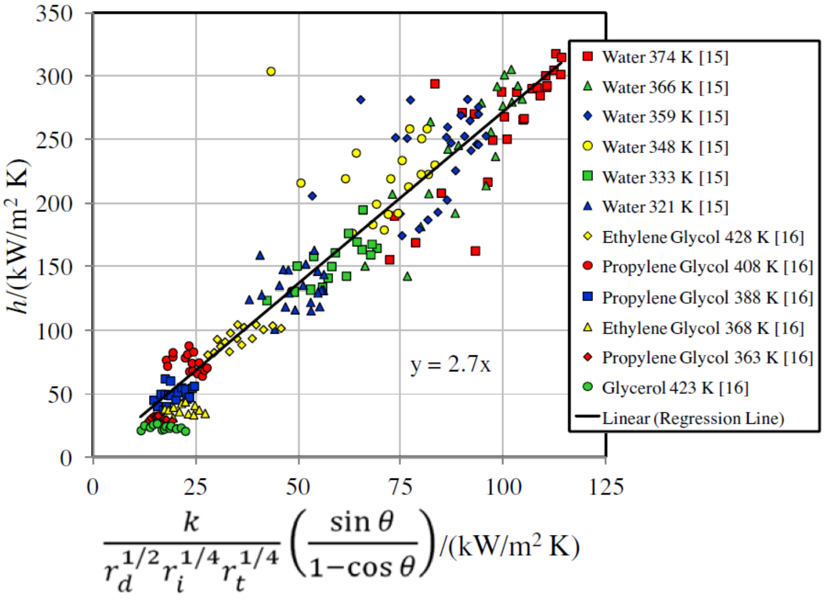

Figure S3: Correlation validation. This figure is taken directly from Bonner (2013) [3] and is Figure 4 in the original paper. This shows the agreement between the correlation used in this study and prior condensation experiments.

S4. SLIPS condensation visualization

The SLIPS were also tested separately in open environment (i.e., outside the AGMD module) to observe the condensation regime. To induce condensation, the surfaces were attached to a thermoelectric cooler via a double-sided tape, in a vertical configuration. The cooler was set to a temperature of 0 °C, which allowed condensation of water droplets on the surface from the surrounding ambient conditions (~21°C and ~38% RH). The condensation process was observed by a lens (VH-Z100R, Keyence) attached to a CCD camera (EO-5023M 2/3" Monochrome, Edmund Optics).

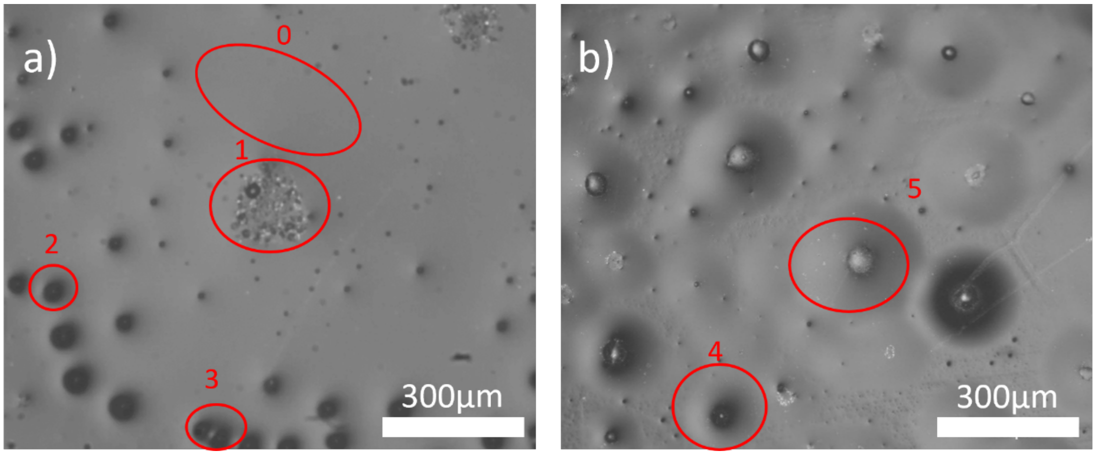

Figure S4: Water droplet on a SLIPS surface a) during condensation, and b) after a single cycle of condensation and evaporation. SLIPS surface morphology before condensation starts is indicated by 0 for reference. Phenomena observed included: lubricant leaking out of the surface and cloaking the condensate (indicated as 1), formation of condensate droplets (indicated as 2), and coalescence of the droplets (indicated as 3), and lubricant forming valleys around droplets (indicated as 4 and 5).

Figure S4 demonstrates SLIPS condensation phenomena such as cloaking of condensate droplets by the lubricant (1), droplet formation (2), and coalescence (3), and lubricant indentation. Cloaking refers to the phenomenon of lubricant leaking out of the surface and forming a layer on top of the condensing water [4]. If the lubricant departs with the droplet, this can impact the quality of water produced through condensation. Water droplets on the surface cause circular indentations on the lubricant layer (4 and 5). This may be due to the contact-line between the three phases. The indentations are bigger for larger droplets.

Table S5: Energy efficiency enhancements at large module lengths using SLIPS compared to filmwise condensation.

AGMD Module Length [m]	Feed Inlet Salinity [g/kg]	Energy Efficiency Enhancements
10	5	2.396%
	35	1.362%
	70	1.427%
	105	0.215%
15	5	2.059%
	35	3.756%
	70	3.847%
	105	2.030%
20	5	1.813%
	35	6.127%
	70	6.294%
	105	3.912%
25	5	1.727%

	35	7.972%
	70	8.324%
	105	5.493%
30	5	1.507%
	35	10.377%
	70	11.016%
	105	7.600%

Reference:

[1] D.E.M.M. Warsinger, J. Swaminathan, L.A. Maswadeh, J.H. Lienhard, J.H. Lienhard V, Superhydrophobic condenser surfaces for air gap membrane distillation, J Memb Sci 492 (2015) 578–587. https://doi.org/10.1016/j.memsci.2015.05.067.

[2] J.W. Rose, Condensation heat transfer fundamentals, Chemical Engineering Research and Design 76 (1998) 143–152. https://doi.org/10.1205/026387698524712.

[3] R.W. Bonner, Correlation for dropwise condensation heat transfer: Water, organic fluids, and inclination, Int J Heat Mass Transf 61 (2013) 245–253. https://doi.org/10.1016/j.ijheatmasstransfer.2012.12.045.

[4] A.A. Günay, S. Sett, Q. Ge, T.J. Zhang, N. Miljkovic, Cloaking Dynamics on Lubricant-Infused Surfaces, Adv Mater Interfaces 7 (2020) 2000983. https://doi.org/10.1002/ADMI.202000983.
